# Dual role for p16 in the metastasis process of HPV positive head and neck cancers

**DOI:** 10.1186/s12943-017-0678-8

**Published:** 2017-06-29

**Authors:** Rüveyda Dok, Mary Glorieux, Karolina Holacka, Marieke Bamps, Sandra Nuyts

**Affiliations:** 1 Department of Oncology, Laboratory of Experimental Radiotherapy, KU Leuven, University of Leuven, 3000 Leuven, Belgium; 2 Department of Radiation Oncology, Leuven Cancer Institute, UH Leuven, Herestraat 49 box 815, 3000 Leuven, Belgium

**Keywords:** HPV, P16, Head and neck cancer, Metastasis, Angiogenesis, Lymphangiogenesis

## Abstract

Several studies show that human papillomavirus (HPV) positive head and neck cancers (HNSCC) are typically characterized by low tumor and high regional node stages, intrinsically indicating high local metastatic potential. Despite this, the distant metastasis rates of HPV positive and negative HNSCC are similar. To date, majority of the studies focus on molecular characterization of HPV positive disease and on treatment outcome. Here we assessed the biological mechanisms of metastasis by combining in vitro and in vivo head and neck carcinoma xenograft models with patient data. We provide experimental evidence for a dual role of p16, a surrogate marker for HPV infections, in the metastasis process of HNSCC. We found that p16 regulates the invasiveness and metastatic potential of HNSCC cells by impairing angiogenesis. In parallel, we found that p16 is regulating the nodal spread by mediating lymphatic vessel formation through the upregulation of integrins. These findings not only provide understanding of the biology of the different dissemination patterns but also suggest that inhibition of lymphangiogenesis in HPV positive cancers and inhibition of angiogenesis in HPV negative cancers can form a treatment strategy against metastasis.

**Electronic supplementary material:**

The online version of this article (doi:10.1186/s12943-017-0678-8) contains supplementary material, which is available to authorized users.

## Background

Head and neck squamous cell carcinoma (HNSCC) is the sixth most common malignancy worldwide with 600,000 cases every year and is associated with high morbidity and mortality [1]. HNSCC patients are divided in two groups according to their etiology, namely high-risk human papilloma virus related (HPV) and the alcohol and tobacco related HNSCC [1–5].

Although recent studies show favorable local and survival outcome of HPV related HNSCC patients, the distant metastasis (DM) rate is similar for HPV positive and negative HNSCC patients [5, 6]. The frequency of distant recurrence in HNSCC is low but with the rarity of locoregional failure, the importance of DM on survival is now more prominent and the leading cause of death in HPV positive patients [6–8]. Even more striking is the unusual dissemination pattern to distal organs like liver and brain in HPV positive HNSCC, compared to HPV negative HNSCC where lung and bone are the most frequent locations for metastasis [9]. Moreover, it is reported that DM in HPV positive HNSCC can still be detected up to 5 years of follow-up [5, 6]. In contrast, DM rates in HPV negative HNSCC are stable after 2 years [5, 6, 9]. HPV positive HNSCC are characterized by low tumor (T) and high regional node (N) stages, intrinsically indicating high local metastatic potential [5, 7, 10]. The significance of T and N stages along with tobacco exposure as factors influencing the risk of recurrence and death in patients with HPV positive HNSCC have been explored in several studies [5, 11–15]. However, the biological assessment behind the unusual and somewhat paradoxical dissemination pattern remains largely unknown. In this paper, we assessed the biological mechanisms behind the clinical presentation of HPV related HNSCC by combining data from in vitro and in vivo HNSCC xenograft models with data of 241 HNSCC patients.

## Main text

### HPV/p16 positive and negative HNSCC show differences in nodal involvement

We determined the HPV and p16 status of 241 oropharyngeal squamous cell carcinoma (OPC) patients treated with (chemo)radiation (c)RT. The human tumor samples were acquired according to protocols approved by the Ethical board of the University Hospitals Leuven (Leuven, Belgium) and implied consent of all the patients were obtained. Clinicopathological data were extracted from patient charts and the mean follow-up time was 4.19 years. HPV positive patients were characterized by lower T stages with 45% (26 out of 58; *p* = 0.025) of HPV positive patients showing T1/2 stage tumors whereas this was 29% (51 out of 175; *p* = 0.025) in the HPV negative group. Moreover, HPV positive tumors showed significantly higher (*p* = 0.014) nodal involvement with 72% (42 out of 58) of HPV positive patients showing N2/N3 tumors whereas this was only 54% (95 out of 175) in the HPV negative group. Although the relationship between p16 status and N stage was not significant, a trend to higher nodal involvement and p16 positivity was seen. Sixty-three% of p16 positive patients showed N2/N3 tumors, whereas this was only 54% in the p16 negative group (Table 1). These results are in concordance with other studies, showing that HPV positive HNSCC patients have higher N and lower T stages [5, 6, 10, 16].

**Table 1:** Correlation between patient characteristics, HPV and p16 status

	*HPV negative*		*HPV positive*		*P*	*p16 negative*		*p16 positive*		*P*
	No.	(%)	No.	(%)		No.	(%)	No.	(%)	
No. of patients										
	175		58			173		68		
Gender					*p* = 0.67^b^					*p* = 0.99^b^
Male	146	83	47	81		143	83	56	82	
Female	29	17	11	19		30	17	12	18	
Age, years					*p* = 0.031^a^					*p* = 0.046^a^
Median (Range)	59	(57–60)	61	(59–65)		59	(57–60)	61	(59–64)	
Smoking history					*p* = 0.001^b^					*p* = 0.004^b^
Never	8	5	15	26		11	6	13	19	
Former	20	11	11	19		22	13	11	16	
Current	126	72	25	43		120	69	35	51	
Unknown	21	12	7	12		20	12	9	13	
Treatment					*p* = 0.68^b^					*p* = 0.15^b^
RT	71	41	19	33		68	39	29	43	
RT + CT	97	55	34	59		100	58	33	49	
RT + EGFR inhibitor	5	3	2	3		3	2	4	6	
Unknown	2	1	3	5		2	1	2	3	
Nodal stage*					*p* = 0.014^b^					*p* = 0.07^b^
N0/N1	80	45	16	28		78	45	25	37	
N2/N3	95	54	42	72		94	54	43	63	
Unknown	2	1	0	0		1	1	0	0	
Tumor stage*					*p* = 0.025^b^					*p* = 0.11^b^
T1/2	51	29	26	45		60	35	22	32	
T3/4	122	70	31	53		112	65	45	66	
unknown	2	1	1	2		1	1	1	1	
Disease stage*					*p* = 0.28^b^					*p* = 0.18^b^
I-II	17	10	3	5		17	10	3	4	
III-IV	157	90	54	93		155	90	65	96	
Unknown	1	1	1	2		1	1	0	0	
HPV										*p* < 0.0001^b^
Negative						150	87	16	24	
Positive						9	5	44	65	
Unknown						14	8	8	12	
p16					*p* < 0.0001^b^					
Negative	150	86	9	16						
Positive	16	9	44	76						
Unknown	9	5	5	9						

*Abbreviations*: *RT* radiotherapy, *CT* chemotherapy, *EGFR* epidermal growth factor

*International Union of Cancer Research 1982 classification; ^a^ANOVA; ^b^chi square test.

As anticipated, low T stages resulted in better distant control (DC) rates with 5-year control rates of 83% and 70% (*p* = 0.04) for T1/T2 and T3/4 tumors respectively (Additional file 1: Figure S1A). N2/3 tumors (5-year DC 70%; *p* = 0.02) showed a higher risk to distant failure compared to N0/1 tumors (5-year DC rate 85%; *p* = 0.02) (Additional file 1: Figure S1B). No statistically significant difference was seen in DC rate between HPV positive and negative disease (5-year DC rate of 82% vs 5-year DC rate of 72% respectively; *p* = 0.20) (Additional file 1: Figure S1C). Although not significant, this 10% difference in DC rates suggests influence of the virus beyond local tumor control. Moreover, it indicates the presence of different dissemination patterns between HPV positive and negative HNSCC.

### p16 represses the invasion and migration capacity of HPV positive HNSCC

**Fig. 1:**
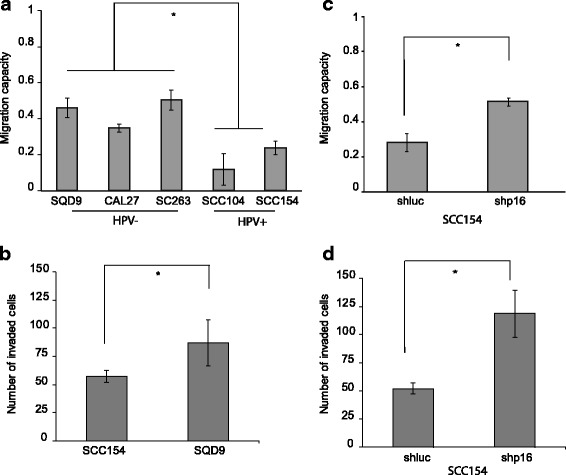
p16 represses the in vitro invasion and migration capacity of HPV positive HNSCC. **a** Migration capacity of HPV negative cells (SQD9, CAL27 and SC263) and HPV positive cells (SCC154, SCC104). **b** Invasion capacity of HPV negative SQD9 cells and HPV positive SCC154 cells. **c** Migration capacity of HPV positive SCC154 cells treated with shRNA for p16 (shp16) and control (shluc). **d** Invasion capacity of HPV positive SCC154 cells treated with shRNA for p16 (shp16) and control (shluc). (**a**-**d**) The result is shown as mean ± SEM of three experiments and *p*-values are calculated by two-sided t-test

To investigate the molecular mechanism behind these potential differences seen in metastasis in our patient cohort, we assessed the in vitro migration and invasion capacity of HNSCC cells. HPV/p16 positive SCC154 and SCC104 cells showed a significant lower migration rate compared to HPV/p16 negative SQD9, CAL27 and SC263 cells (Fig. 1a). In agreement with the migration assay, HPV/p16 positive cells showed reduced invasion abilities compared to the HPV/p16 negative cells (Fig. 1b).

The tumor suppressor p16 is a well-known cell cycle regulator and a good surrogate marker for HPV positive HNSCC [2, 3, 15]. Moreover, recent data ascribe a broader role for p16, including a role in migration and repression of angiogenesis [17–20]. Therefore, we also examined the influence of p16 on the migration and invasion capacity of HPV positive SCC154 cells manipulated with short hairpin RNA (shRNA) for p16 (shp16) and control shRNA (shluc). Downregulation of p16 expression increased migration and invasion capacities of the SCC154 cells (Fig. 1c and d). These data verify the presence of differences in dissemination patterns between HPV positive and negative HNSCC and suggest an active role of p16 in the metastatic cascade.

### p16 suppresses migration and invasion through angiogenesis in HPV positive HNSCC

It is well known that angiogenesis, which is actively sustained in cancer cells by pro-angiogenic factors such as vascular endothelial growth factor A (VEGFA), is a conduit for cancer cell spread and metastasis and promotes aggressive tumor progression [18, 21, 22]. Moreover, the negative correlation between HPV/p16 and VEGFA is previously described in HNSCC [18]. Therefore, we assessed the relation between HPV, p16 and VEGFA in our patient cohort. HPV positive patients showed significantly lower VEGFA expression with 54% (28 out of 51) of patients showing no or low VEGFA expression compared to 32% (53 out of 161) in HPV negative group (Table 2). Although the negative correlation between VEGFA and p16 was less pronounced, a trend to significance was seen (Table 2).

**Table 2:** Correlation between VEGF expression, HPV and p16 in HNSCC patients

	VEGF low		VEGF high		*P*
	No.	(%)	No.	(%)	
HPV					*p*=0.005^b^
Negative	53	65	108	82	
Positive	28	35	23	18	
p16					*p*=0.07^b^
Negative	53	66	99	77	
Positive	27	34	29	23	

^b^chi square test

**Fig. 2:**
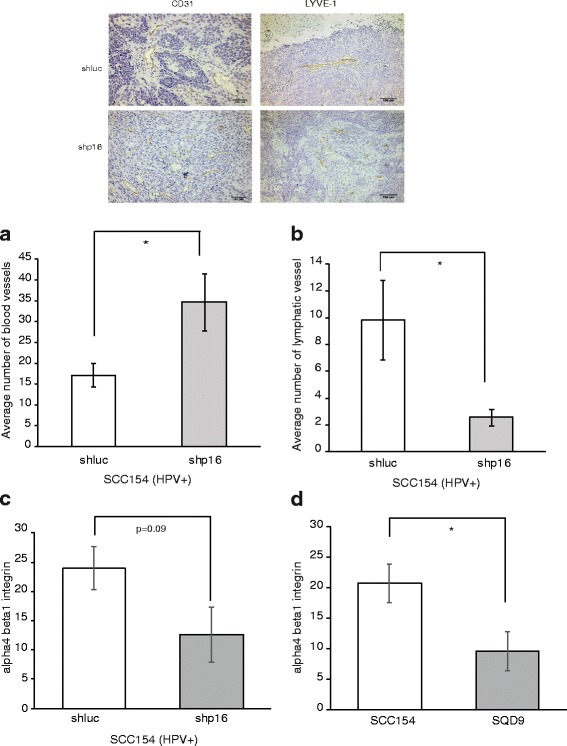
Dual role of p16 in dissemination of HNSCC. **a** Blood vessel formation in SCC154 shp16 and SCC154 shluc xenograft mouse models assessed by CD31 staining (above the graph); *n* = 5. **b** Average number of lymphatic vessel formation in SCC154 shp16 and SCC154 shluc xenograft mouse models assessed by LYVE-1 staining (above the graph); *n* = 5. **c** Average score of alpha4 beta1 integrin staining in SCC154 shp16 and SCC154 shluc mouse xenograft models; *n* = 5. **d** Average score of alpha4 beta1 integrin staining in HPV positive SCC154 (*n* = 7) and HPV negative SQD9 (*n* = 10) mouse xenograft models. **a**-**d** *P*-values are calculated by two-sided t-test

We further investigated the relation between angiogenesis and p16 with in vivo xenografts injected with SCC154 HPV positive cells manipulated with shp16 or shluc. A higher number of blood vessels was detected in mice tumors with low p16 expression compared to control tumors (Fig. 2a). Furthermore, the increase in vascularization resulted in lower necrosis, higher number of mitotic cells and growth advantage of shp16 expressing tumors (Additional file 2: Figure S2A-C). We could not verify the involvement of p16 in suppression of metastasis in vivo due to the absence of a metastatic animal model. However, the growth advantage and the increased vascularization seen in shp16 mice tumors can explain the frequent occurrence of advanced T stages in HPV negative HNSCC patients.

### p16 stimulates lymphangiogenesis and nodal spread in HPV positive HNSCC

These results still do not explain the occurrence of the similar distant relapse rates in HPV/p16 positive and negative HNSCC patients and certainly do not explain the highly significant association between HPV positivity and nodal involvement described in our and several other studies [5, 6, 10, 16]. This is especially important since it implicates the presence of a high local metastatic potential in HPV positive HNSCC. Interestingly lymphangiogenesis, like angiogenesis, promotes tumor metastasis by inducing the growth of new lymphatic vessels within the tumor and by enhancing cell trafficking to lymph nodes. Moreover, increased lymphatic vessel density in tumors is associated with increased metastasis to lymph nodes. [21, 23, 24]. Therefore, we assessed the lymphatic vessel formation by homologue lymphatic vessel hyaluronan (LYVE-1) immunostaining in shluc and shp16 mice tumors. We found that p16 suppression resulted in lower lymphatic vessel density in HPV/p16 positive xenografts suggesting a dual role of p16 in metastasis (Fig. 2b).

To understand the function of p16, we focused on integrins as these proteins are accepted as key regulators of angiogenesis and lymphangiogenesis [21, 23–27]. Moreover, the binding of p16 to integrins and loss of cell spread is previously documented [20, 28]. Integrins such as alpha4 beta1 are important modulators of lymphangiogenesis [21, 23, 24]. Therefore, we assessed the presence of alpha4 beta1 integrin in SCC154 shluc and SCC154 shp16 mice tumors. Although not statistically significant (*p* = 0.09), shluc tumors showed a higher percentage of integrin compared to shp16 tumors (Fig. 2c). In line with SCC154 shRNA xenografts, tumors from HPV/p16 positive SCC154 xenografts showed higher percentage of alpha4 beta 1 integrin compared to tumors from HPV/p16 negative SQD9 xenografts (Fig. 2d).

## Conclusion

Taken together, we provide experimental evidence for a dual role of p16 in the metastasis process of HNSCC. We show that p16 on the one hand regulates vascular invasiveness and growth of the tumor cells by inhibiting angiogenesis; on the other hand, it stimulates nodal spread by enhancing lymphangiogenesis. These findings provide us a better understanding of the molecular principles underlying the dissemination patterns and clinical presentation of HNSCC. Importantly, it also opens different treatment opportunities for metastasis in HNSCC by the inhibition of vascularization (e.g. anti-angiogenic drugs) in HPV negative cancers and by the inhibition of lymphangiogenesis (e.g. alpha4 antagonist) in HPV positive cancers. However, further preclinical and clinical studies are necessary to confirm these results and to investigate the utility and specificity of these treatment approaches.

## Additional files

Additional file 1: Figure S1. HPV/p16 positive and negative HNSCC patients show differences in nodal involvement. (A) Distant control (DC) rates in HNSCC patients with different T stages presented by Kaplan-Meier curves. (B) Distant control (DC) rates in HNSCC patients with different N stages presented by Kaplan-Meier curves (C) Distant control (DC) rates in HNSCC patients with different HPV status by Kaplan-Meier curves. (A-C) *P* values are determined by log-rank tests. (PDF 411 kb)

Additional file 2: Figure S2. Dual role of p16 in dissemination of HNSCC. (A) Average tumor necrosis in SCC154 shp16 and SCC154 shluc xenograft mouse models; *n* = 5. (B) Average number of mitotic cells in SCC154 shp16 and SCC154 shluc xenograft mouse models; *n* = 5. (C) Average tumor volume of SCC154 shp16 and SCC154 shluc xenograft mouse models assessed by caliper measurements; *n* = 5. (A-C) *P*-values are calculated by two-sided t-test. (PDF 351 kb)

Additional file 3: Supplementary material and methods. Detailed description of the material and methods. (DOCX 19 kb)

## Abbreviations

(c)RT: (Chemo)radiation therapy
CT: Chemotherapy
DC: Distant control
DM: Distant metastasis
EGFR: Epidermal growth factor
HNSCC: Head and neck squamous cell carcinoma
HPV: Human papillomavirus
LYVE-1: Homologue lymphatic vessel hyaluronan receptor
N: Node
OPC: Oropharyngeal squamous cell carcinoma patients
shRNA: short hairpin RNA
shluc: shRNA luciferase
shp16: shRNA p16
T: Tumor
VEGFA: Vascular endothelial growth factor A

## References

[1] Jemal A, Siegel R, Ward E, Hao Y, Xu J, Thun MJ (2009). Cancer statistics, 2009. CA Cancer J Clin.

[2] Lassen P (2010). The role of human papillomavirus in head and neck cancer and the impact on radiotherapy outcome. Radiother Oncol.

[3] Leemans CR, Braakhuis BJ, Brakenhoff RH (2011). The molecular biology of head and neck cancer. Nat Rev Cancer.

[4] Marur S, Forastiere AA (2008). Head and neck cancer: changing epidemiology, diagnosis, and treatment. Mayo Clin Proc.

[5] Ang KK, Harris J, Wheeler R, Weber R, Rosenthal DI, Nguyen-Tan PF (2010). Human papillomavirus and survival of patients with oropharyngeal cancer. N Engl J Med.

[6] O'Sullivan B, Huang SH, Siu LL, Waldron J, Zhao H, Perez-Ordonez B (2013). Deintensification candidate subgroups in human papillomavirus-related oropharyngeal cancer according to minimal risk of distant metastasis. J Clin Oncol.

[7] Spector ME, Gallagher KK, Bellile E, Chinn SB, Ibrahim M, Byrd S (2014). Patterns of nodal metastasis and prognosis in human papillomavirus-positive oropharyngeal squamous cell carcinoma. Head Neck.

[8] Spector ME, Gallagher KK, Light E, Ibrahim M, Chanowski EJ, Moyer JS (2012). Matted nodes: poor prognostic marker in oropharyngeal squamous cell carcinoma independent of HPV and EGFR status. Head Neck.

[9] Huang SH, Perez-Ordonez B, Liu FF, Waldron J, Ringash J, Irish J (2012). Atypical clinical behavior of p16-confirmed HPV-related oropharyngeal squamous cell carcinoma treated with radical radiotherapy. Int J Radiat Oncol Biol Phys.

[10] O'Sullivan B, Huang SH, Perez-Ordonez B, Massey C, Siu LL, Weinreb I (2012). Outcomes of HPV-related oropharyngeal cancer patients treated by radiotherapy alone using altered fractionation. Radiother Oncol.

[11] Fakhry C, Westra WH, Li S, Cmelak A, Ridge JA, Pinto H (2008). Improved survival of patients with human papillomavirus-positive head and neck squamous cell carcinoma in a prospective clinical trial. J Natl Cancer Inst.

[12] Gillison ML, Zhang Q, Jordan R, Xiao W, Westra WH, Trotti A (2012). Tobacco smoking and increased risk of death and progression for patients with p16-positive and p16-negative oropharyngeal cancer. J Clin Oncol.

[13] Lowy DR, Munger K (2010). Prognostic implications of HPV in oropharyngeal cancer. N Engl J Med.

[14] Maxwell JH, Kumar B, Feng FY, Worden FP, Lee JS, Eisbruch A (2010). Tobacco use in human papillomavirus-positive advanced oropharynx cancer patients related to increased risk of distant metastases and tumor recurrence. Clin Cancer Res.

[15] Rischin D, Young RJ, Fisher R, Fox SB, Le QT, Peters LJ (2010). Prognostic significance of p16INK4A and human papillomavirus in patients with oropharyngeal cancer treated on TROG 02.02 phase III trial. J Clin Oncol.

[16] Quon H, Forastiere AA (2013). Controversies in treatment deintensification of human papillomavirus-associated oropharyngeal carcinomas: should we, how should we, and for whom?. J Clin Oncol.

[17] Al-Ansari MM, Hendrayani SF, Tulbah A, Al-Tweigeri T, Shehata AI, Aboussekhra A (2012). p16INK4A represses breast stromal fibroblasts migration/invasion and their VEGF-a-dependent promotion of angiogenesis through Akt inhibition. Neoplasia.

[18] Baruah P, Lee M, Wilson PO, Odutoye T, Williamson P, Hyde N (2015). Impact of p16 status on pro- and anti-angiogenesis factors in head and neck cancers. Br J Cancer.

[19] Chen YW, Chu HC, Ze-Shiang L, Shiah WJ, Chou CP, Klimstra DS (2013). p16 stimulates CDC42-dependent migration of hepatocellular carcinoma cells. PLoS One.

[20] Romagosa C, Simonetti S, Lopez-Vicente L, Mazo A, Lleonart ME, Castellvi J (2011). Ramon y Cajal S: p16(Ink4a) overexpression in cancer: a tumor suppressor gene associated with senescence and high-grade tumors. Oncogene.

[21] Avraamides CJ, Garmy-Susini B, Varner JA (2008). Integrins in angiogenesis and lymphangiogenesis. Nat Rev Cancer.

[22] Carmeliet P (2005). Angiogenesis in life, disease and medicine. Nature.

[23] Garmy-Susini B, Makale M, Fuster M, Varner JA (2007). Methods to study lymphatic vessel integrins. Methods Enzymol.

[24] Jin H, Varner J (2004). Integrins: roles in cancer development and as treatment targets. Br J Cancer.

[25] Brooks PC, Stromblad S, Klemke R, Visscher D, Sarkar FH, Cheresh DA (1995). Antiintegrin alpha v beta 3 blocks human breast cancer growth and angiogenesis in human skin. J Clin Invest.

[26] Friedlander M, Theesfeld CL, Sugita M, Fruttiger M, Thomas MA, Chang S (1996). Involvement of integrins alpha v beta 3 and alpha v beta 5 in ocular neovascular diseases. Proc Natl Acad Sci U S A.

[27] Hirakawa S, Kodama S, Kunstfeld R, Kajiya K, Brown LF, Detmar M (2005). VEGF-A induces tumor and sentinel lymph node lymphangiogenesis and promotes lymphatic metastasis. J Exp Med.

[28] Fahraeus R, Lane DP (1999). The p16(INK4a) tumour suppressor protein inhibits alphavbeta3 integrin-mediated cell spreading on vitronectin by blocking PKC-dependent localization of alphavbeta3 to focal contacts. EMBO J.

