# Supplementary figures and images for: Dual role for p16 in the metastasis process of HPV positive head and neck cancers
Source: Mol Cancer. 2017 Jun 29;16:113. doi: 10.1186/s12943-017-0678-8 (PMC5492443; doi:10.1186/s12943-017-0678-8)

A

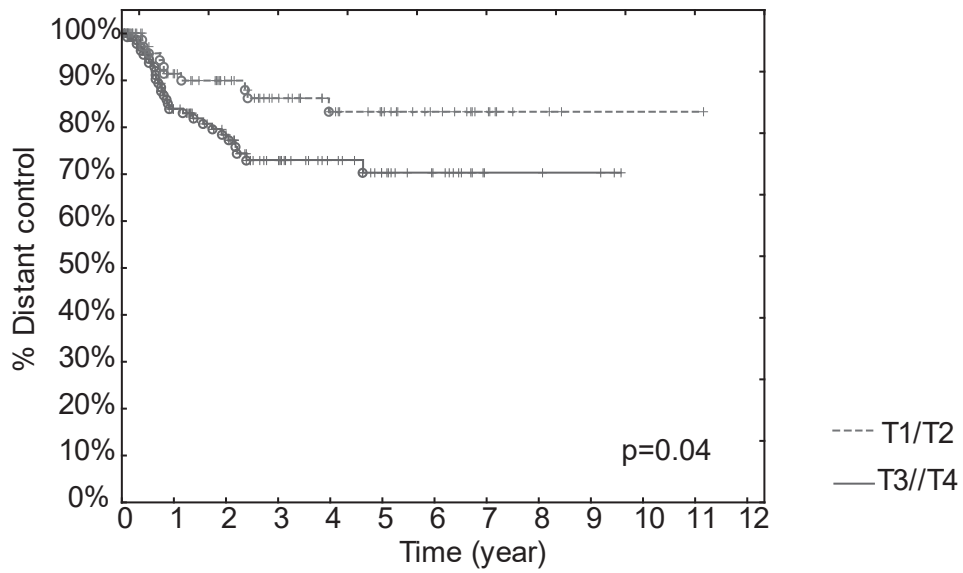

B

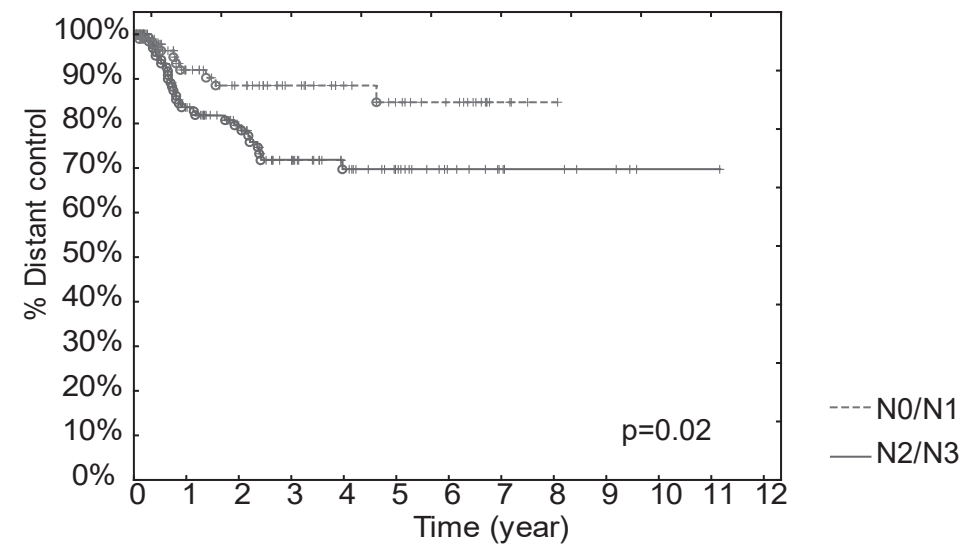

C

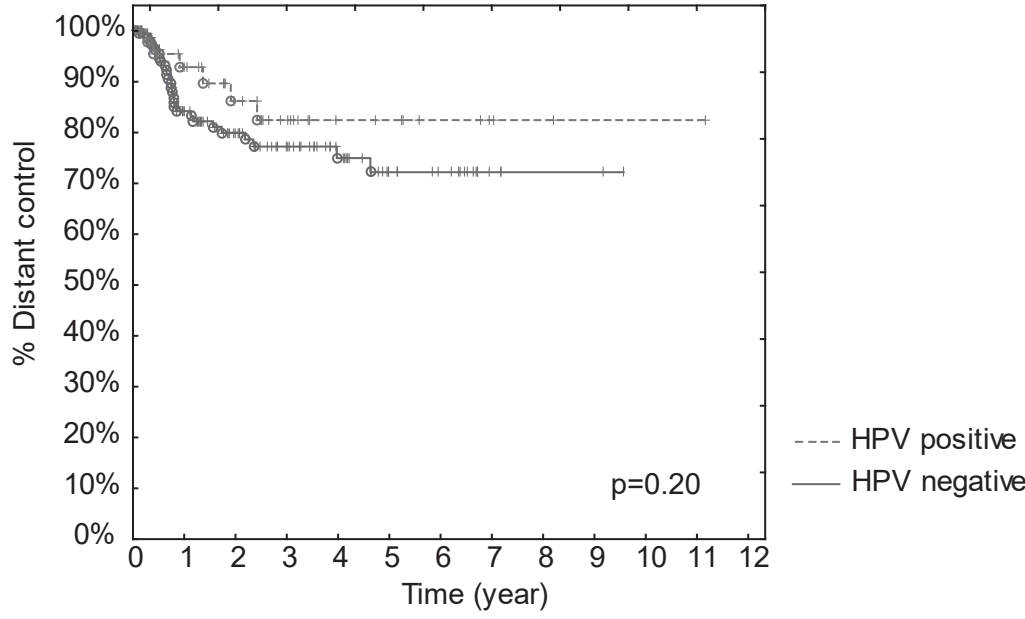

Supplement: Supplementary file 1 — HPV/p16 positive and negative HNSCC patients show differences in nodal involvement. (A) Distant control (DC) rates in HNSCC patients with different T stages presented by Kaplan-Meier curves. (B) Distant control (DC) rates in HNSCC patients with different N stages presented by Kaplan-Meier curves (C) Distant control (DC) rates in HNSCC patients with different HPV status by Kaplan-Meier curves. (A-C) P values are determined by log-rank tests. (PDF 411 kb) [file 12943_2017_678_MOESM1_ESM.pdf]

A

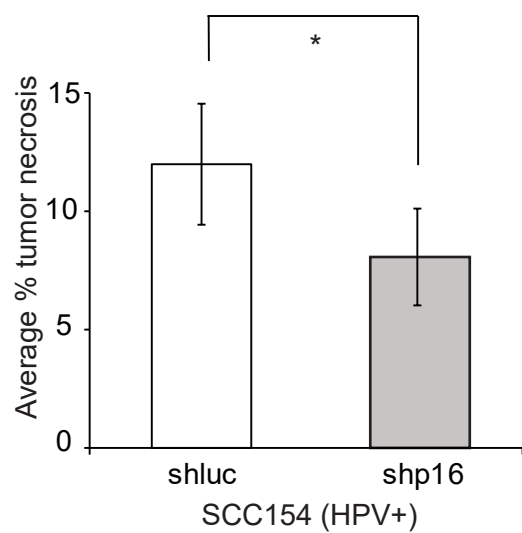

B

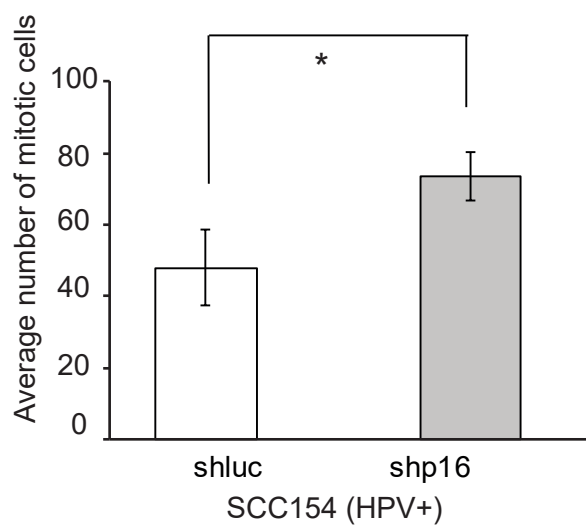

C

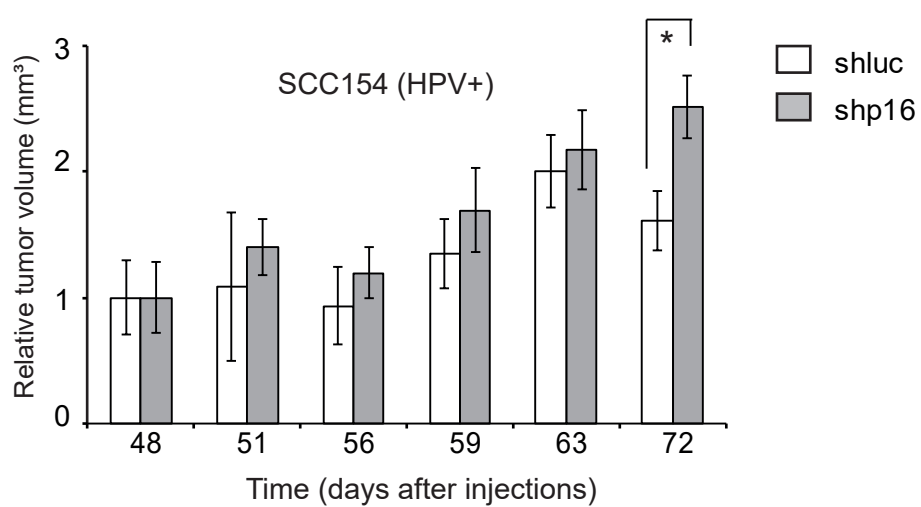

Supplement: Supplementary file 2 — Dual role of p16 in dissemination of HNSCC. (A) Average tumor necrosis in SCC154 shp16 and SCC154 shluc xenograft mouse models; n = 5. (B) Average number of mitotic cells in SCC154 shp16 and SCC154 shluc xenograft mouse models; n = 5. (C) Average tumor volume of SCC154 shp16 and SCC154 shluc xenograft mouse models assessed by caliper measurements; n = 5. (A-C) P-values are calculated by two-sided t-test. (PDF 351 kb) [file 12943_2017_678_MOESM2_ESM.pdf]
