# Supplementary material for: Dual role for p16 in the metastasis process of HPV positive head and neck cancers
Source: Mol Cancer. 2017 Jun 29;16:113. doi: 10.1186/s12943-017-0678-8 (PMC5492443; doi:10.1186/s12943-017-0678-8)
Supplement: Supplementary file 3 — Supplementary material and methods. Detailed description of the material and methods. (DOCX 19 kb) [file 12943_2017_678_MOESM3_ESM.docx]

**Methods**

**Study cohort and immunohistochemistry of patient biopsies**

The study cohort consisted out of 241 oropharyngeal squamous cell carcinoma (OPC) patients, diagnosed between 2000 and 2010 and treated with (chemo)radiation (c)RT. The mean follow-up time was 4.19 years. The human tumor samples were acquired according to protocols approved by the Ethical board of the University Hospitals Leuven (Leuven, Belgium) and implied consent of all the patients were obtained. Clinicopathological data were extracted from patient charts. P16 and HPV status of the patient samples were determined by p16 immunohistochemistry and HPV polymerase chain reaction (PCR). The tumors were classified as p16 positive in case of strong, diffuse nuclear staining in more than 10% of HNSCC cells as previously described [16, 17]. Vascular endothelial growth factor A (VEGFA) (SC-152, Santa Cruz) expression was classified in three groups according to staining intensity with the lowest class showing no or very low staining of VEGFA and the highest class showing maximal immunoreactivity [18].

**Cell lines and reagents**

The HPV/p16 negative HNSCC cells SQD9, CAL27 and SC263 (gift from Dr. A. Begg from the Netherlands Cancer Institute) were cultured in Dulbecco minimum essential media (DMEM) supplemented with 10% fetal bovine serum and 1% sodium pyruvate (Life technologies). The HPV/p16 positive HNSCC cells SCC154 (DSMZ) and SCC104 (gift from Dr. C. Thomas from Michigan University) were cultured and maintained in minimum essential media (MEM) supplemented with 10% fetal bovine serum, 1% L-glutamine and 1% non-essential amino acids. Lentiviral shRNAs against luciferase (shluc) and p16 (shp16; TRCN0000265833) were purchased from Sigma-Aldrich.

**In vitro migration and invasion assay**

Migration assay kit (Platypus Oris®) was used to assess the migration according to the manufacturer’s instructions. Differences between the areas without migrated cells were measured (Image J) at 0 and 24 hours. Matrigel (BD biosciences) coated transwell filters (Corning Costar Corp.) were used to investigate the invasive properties of the HNSCC cell lines. HNSCC cells were seeded in serum free media in the upper chambers and serum-containing media was used in the lower chambers as an attractant. After 24 hours, invaded cells were stained with crystal violet and counted under the microscope (Olympus). All experiments were performed in triplicates.

**In vivo experiments**

Female NMRI-nu/nu mice were subcutaneously injected with SCC154 shluc (8 million), SCC154 shp16 (8 million), SCC154 (8million) or SQD9 (2.5 million) cells in each flank. Different number of cells were inoculated because of differences in the hit-rates of the tumors. The tumor volume was assessed by caliper measurement. The body weight and health of the mice were monitored daily. All experiments were performed according to the Ethical committee of KU Leuven (P141/2013).

For immunohistochemistry experiments paraffin-embedded mice tissue blocks were cut 4µm and stained for p16 (G175-405, BD Pharmingen), CD31 (SZ31, Dianova), homologue lymphatic vessel hyaluronan (LYVE-1) (NB600-1008, Novus) and alpha4 beta1 integrin (AP-MAB0865, Abcam). Immunodetection was performed with EnVision HRP anti-Mouse (Dako) for p16, EnVision HRP Anti-Rabbit (Dako) for LYVE-1, impress reagent kit anti-RAT (vector lab) for alpha4 beta1 integrin and biotinylated secondary antibody goat anti-rat IgG (eBioscience) for CD31. The average number of mitotic cells and percentage of necrotic tumor tissue were assessed in hematoxylin stained sections. Scoring of mitosis, necrosis, CD31 and LYVE-1 was performed in 5 fields with magnification of 200X. For the analysis of alpha4 beta1 integrin staining a histoscore was calculated by multiplying the percentage with the intensity of the staining.

**Statistical analysis**

Survival rates of HNSCC patients were determined by Kaplan-Meier estimates and log-rank test was used to compare survival intervals. Distant control of HNSCC patients was defined as time between start of radiotherapy and distant recurrence of disease. Differences between different groups were assessed by a Chi-square test in case of categorical predictors whereas the one-way analysis of variance was used in cases of continuous predictors. For the in vitro and in vivo xenograft experiments, student t-test was used. All statistical analyses were performed 2 sided and were considered statistically significant for p≤0.05. Statistica 12 software was used.
